# A Genomics Resource for 12 Edible Seaweeds to Predict Seaweed-Secreted Peptides with Potential Anti-Cancer Function

**DOI:** 10.3390/biology11101458

**Published:** 2022-10-04

**Authors:** Yining Liu, Scott F. Cummins, Min Zhao

**Affiliations:** 1 The School of Public Health, Institute for Chemical Carcinogenesis, Guangzhou Medical University, Guangzhou 510180, China; 2 Seaweed Research Group, University of the Sunshine Coast, Maroochydore, QLD 4558, Australia; 3 School of Science, Technology and Engineering, University of the Sunshine Coast, Maroochydore, QLD 4558, Australia

**Keywords:** edible seaweed, nutraceutical, genome database, protein interaction, anti-cancer

## Abstract

**Simple Summary:**

Edible seaweed, also known as sea vegetables, are macroalgae that can be eaten and used in cooking. They contain enriched bioactive compounds such as polysaccharides, which have a variety of functions such as antioxidants, anti-aging, and immune regulation. This study was a voyage of biological discovery that sought to thoroughly examine all of the DNA (known as a genome) of 12 edible seaweeds. The collected genomic sequence data are publicly accessible as a critical functional reference for the discovery of bioactive products and genome-assisted breeding for edible seaweeds. Secreted proteins frequently circulate throughout the body and, thus, have access to the majority of organs and tissues in animals. We investigate the potential secreted short peptides in seaweed using a comparative analysis of protein families that interact with human cancer genes. Some of the secreted proteins may be therapeutic agents interacting with surface receptors in human cells due to their circulating function. In summary, our data integration predicted the first shortlist of secreted peptides of edible seaweeds, which may provide novel information about anti-tumour mechanisms, thus accelerating the study of seaweed biology and improving seaweed nutraceutical practice.

**Abstract:**

Seaweeds are multicellular marine macroalgae with natural compounds that have potential anticancer activity. To date, the identification of those compounds has relied on purification and assay, yet few have been documented. Additionally, the genomes and associated proteomes of edible seaweeds that have been identified thus far are scattered among different resources and with no systematic summary available, which hinders the development of a large-scale omics analysis. To enable this, we constructed a comprehensive genomics resource for the edible seaweeds. These data could be used for systematic metabolomics and a proteome search for anti-cancer compound and peptides. In brief, we integrated and annotated 12 publicly available edible seaweed genomes (8 species and 268,071 proteins). In addition, we integrate the new seaweed genomic resources with established cancer bioinformatics pipelines to help identify potential seaweed proteins that could help mitigate the development of cancer. We present 7892 protein domains that were predicted to be associated with cancer proteins based on a protein domain–domain interaction. The most enriched protein families were associated with protein phosphorylation and insulin signalling, both of which are recognised to be crucial molecular components for patient survival in various cancers. In addition, we found 6692 seaweed proteins that could interact with over 100 tumour suppressor proteins, of which 147 are predicted to be secreted proteins. In conclusion, our genomics resource not only may be helpful in exploring the genomics features of these edible seaweed but also may provide a new avenue to explore the molecular mechanisms for seaweed-associated inhibition of human cancer development.

## 1. Introduction

According to World Health Organization estimates, there were approximately 19.3 million new cancer cases in 2020, of which 10.06 million were in males and 9.23 million were in females [1]. Based on this, one in five people worldwide will have cancer in their lifetime. As the global population grows and life expectancy increases, cancer will become more common, and the global cancer burden will further increase, especially in low- and middle-income countries. Currently, 70% of patients who die from cancer live in low-to middle-income countries. In general, cancer and cancer metastasis may result from dysfunction of one of the numerous cellular functions involving thousands and millions of molecular interactions. Some common molecular mechanisms could be revealed from the investigation of differentially expressed genes between normal and cancer tissues. For instance, defective tumour suppressor genes (TSGs) [2] and hyperactive oncogenes (OCGs) [3] can be driving factors for cancer initiation and progression.

Many cancer-associated deaths can be avoided by choosing a healthy diet such as fruits and vegetables [4]. Additionally, edible seaweeds (as a food or food additive) have enormous potential, and there are more than one hundred different types of edible seaweed. The consumption of edible seaweed is widespread in tropical South Asia and Southeast Asia, while it is relatively limited in Western societies. The impact of edible seaweed on cancer prevention has been widely studied. For instance, the cancer mortality rates of Okinawa Island inhabitants, where kelp and wakame are commonly eaten, have always been the lowest in Japan [5]. This is particularly relevant to stomach, colon, and rectal cancers [6,7,8]. As such, seaweed-derived natural products have been investigated, including the macromolecular polysaccharide fucoidan [9]; dieckol [10]; matrix metalloproteinase inhibitors [11]; and algae fucoxanthin and its deacetylated product, fucoxanthinol [12]. Seaweed proteins and peptides (derived from a precursor protein) rich in essential amino acids have also been explored, where the biological effects described include antimicrobial and antioxidant activity (reviewed by [13]). Besides the potential nutraceutical preventative benefits, seaweed products may also be applied as cancer therapeutic, such as inhibition of cancer cell growth and invasion. For this, knowledge of the compounds (small molecule or protein), as well as the ability to purify and test homogenous preparations are important. Therefore, the pathway from identification to clinical evaluation can be a long-term process [14]. 

In general, genome, proteomics, and metabolomics have all made significant contributions to our understanding of seaweed biology. Although the above experimental evidence links many seaweed bioactive compounds with anti-cancer effects, there is still a lack of a big picture for seaweed biology. With the advance in high-throughput sequencing technologies, many seaweed genomes have been sequenced, which has provided us with an encyclopedia of molecular functions [15,16,17,18,19,20,21,22]. For example, a chromosome-level assembly of edible brown alga *Undaria pinnatifida* was generated by using PacBio and Hi-C technologies [20].

As well known, the cell is far more complicated than the sum of its genes, proteins, or metabolites. In general, the anti-cancer effects are the activity of all those components and their interactions that add up to those edible seaweed. However, these data were dispersed throughout the literature and a systematic review is lacking. To explore the complexity of seaweed, we aim to collect genome and proteome data for edible seaweeds, which will advance our knowledge about how these compounds may safeguard cancers. 

In addition, these omics data may provide reliable opportunities to explore the molecular interactions between seaweed compounds and cancer cells. Once identified, cross-species comparisons can be used to determine similarities and differences between seaweeds and, therefore, to predict their anti-cancer effects. Given this, we have carried out the first systematic data integration and published it as a web database for genome-level molecular interaction investigation in an attempt to identify all possible molecular interactions theoretically related to cancer biology. Specifically, we aimed to computationally connect seaweed protein-coding genes with known TSG and OCG proteins in humans. We explored (1) the interactional preference of seaweed proteins with tumour suppressor and oncogene proteins, (2) common interaction patterns between the 12 seaweeds, and (3) common cancer proteins with the most abundant interactions with seaweed proteins.

## 2. Results

### 2.1. Computational Framework to Predict Putative Interactions between Seaweed Genomes and Human Cancer Genes

**Figure 1:**
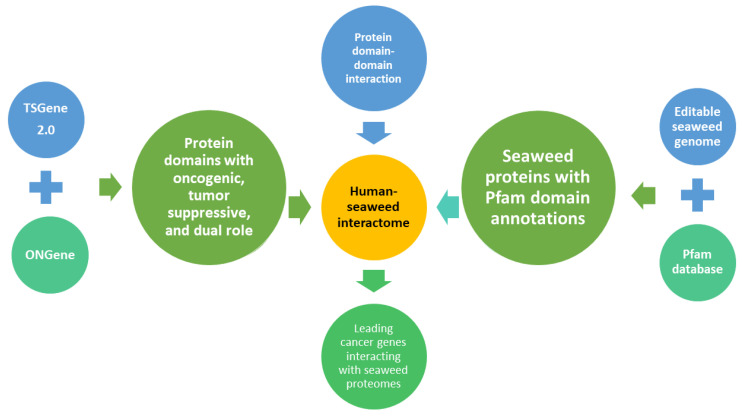
A computational workflow to construct a putative interactome between cancer-related and seaweed proteins based on known interactions between functional protein domains. Specifically, cancer-related genes in this study refer to known tumour suppressor genes and oncogenes. The seaweed protein sequences were derived from 12 seaweed genomes. We then annotated all human cancer genes and edible seaweed proteins through the hidden Markov modes profile of the Pfam database. Finally, potential links between seaweed and cancer proteins were built through the functional domain–domain interactions.

First, we conducted an extensive literature review to search for publicly available seaweed genomes. As summarised in Figure 1, seaweed protein-coding genes were annotated and human genes with protein domain information were used to link the seaweed proteins with human cancer genes based on well-known protein domain–domain interactions [23]. In total, we found 13 publicly available seaweed genomes; however, the *Macrocystis pyrifera* (giant kelp) genome was highly fragmented, with a contig N50 of only 2573 bp. Therefore, we excluded this assembly and focused on the remaining 12 genomes (Table 1). Among these 12 genomes, 4 were different strains of the same species, *Cladosiphon okamuranus* [14], an Okinawan seaweed most commonly known as the Japanese brown algae Mozuku. One of the most promising natural products with anti-cancer and anti-coagulant functions is the fucoidan from Mozuku. As a sulphated water-soluble polysaccharide, fucoidan also has beneficial effects against chemotherapeutic agents and radiation toxicity. In addition, two genome cultivars of *Saccharina japonica* were used, which had been sequenced to explore polysaccharide biosynthesis and iodine antioxidation [22]. The remaining six seaweed genomes were from distinct genus’s and presented divergent genetic contents.

Among the 12 genomes, 5 had existing predicted protein-coding proteomes available. The remaining (*Undaria pinnatifida*, *Ulva mutabilis,* and two strains of *Saccharina japonica*) did not, and therefore, we utilised an in-house gene prediction pipeline to predict the protein sequences. All proteins from the 12 seaweed genomes were annotated with known protein family/domain information from the most updated Pfam database [23]. By applying an annotation pipeline to the 12 edible seaweed genome data, we identified 268,071 protein sequences.

To investigate the potential interaction of seaweed protein with cancer-related proteins at the molecular level, we focused on two of the most crucial cancer gene groups: TSGs and OCGs. It should be noted that some genes have dual roles, meaning those genes could function as TSGs or OCGs, depending on the tissue specificity or interacting partners [2,31]. In the current TSGene 2.0 [2] and ONGene [3] databases, there are 1217 TSGs (1018 protein-coding TSGs) and 803 OCGs (698 protein-coding OCGs), while 129 genes have dual roles. To determine the oncogenic functions of the protein interacting domains, we mapped all of the protein-coding TSGs and OCGs to 1255 Pfam entries, which led to 335 OCG-specific and 621 TSG-specific domains. In addition, we found 299 Pfam domains with both TSGs and OCGs association, which were defined as dual role Pfam domains (Table S1). These Pfam annotated TSGs/OCGs and genome-derived seaweed proteins provided the resource to build an interacting relationship based on the high-quality domain-domain interactions data predicted from the DOMINE database [32]. The output was a connected putative interactome that comprised the human TSGs/OCGs and seaweed proteomes. 

To further group the interactions, Pfam domains were classified as TSG-specific, OCG-specific, and dual role, which defined the putative interaction function. This framework was most helpful in identifying common domain–domain interactions across the 12 putative interactomes presented. In this way, we could further determine if any molecular interactions were shared in multiple seaweeds. In addition, the outcome was helpful in prioritising the top-interacting cancer proteins that may represent candidate pharmaceutical targets associated with patient survival.

### 2.2. The Online Data Sharing Interface for the Putative Interactomes and Overall Statistics for Human TSGs/OCGs and Seaweed Putative Interactomes

Using our computational framework, 12 putative interactomes were constructed between human TSGs/OCGs and edible seaweeds based on protein domains. In addition, this provided a basis to explore the commonness and uniqueness among various seaweed divisions across brown, green, and red seaweeds. 

Since those edible algae genomes were published in recent years, many underlying biomedical functions for animal and clinical health is largely unknown. However, only four species have the assembly without any protein information. This may obstruct further data mining for researchers with no genomic and bioinformatics skills. Considering that our data are valuable for the community to explore potential natural products and other pharmaceutical applications, we built an online web interface to share all of the protein sequences and annotations.

**Figure 2:**
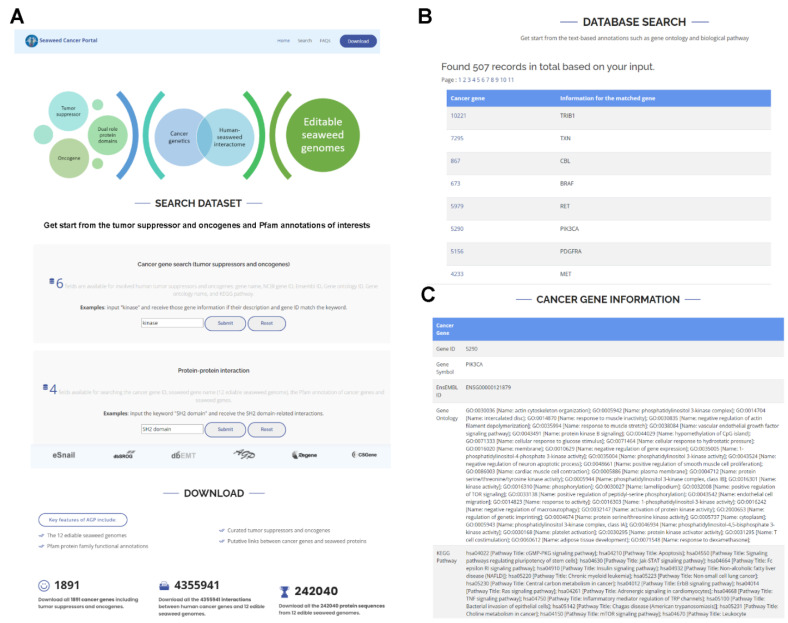
A web browsing interface for the interactome between human cancer genes and twelve edible seaweed genomes. (**A**) The homepage includes the data search interface, and the data download links. (**B**) The search result will guide the user to each cancer-related gene page. (**C**) On each gene page, we provide basic annotation information, such as gene ontology and KEGG pathway of the cancer genes.

As shown in Figure 2A, the web interface provides access to all 268,071 protein sequences from the twelve edible seaweed genomes. From the web page, we can browse the distribution of related genes in each species according to each Pfam protein family. We also organised all processed genetic interaction data into text files for users to download. In practice, the amino acid sequences in FASTA format are shared in the download section. 

In addition, all 1891 non-redundant cancer genes including both TSGs and OCGs are searchable (Figure 2B). At the same time, we can also search our predicted interactions by any protein family of interests. In the cancer gene page (Figure 2C), the user could browse the basic annotations such as gene ontology and KEGG pathways. All of the 4,355,941 putative interactions between cancer genes and algae proteins are shared for bulk download, which could be used for follow-up data mining. Based on these proceeded data, users can explore not only the links between algae genomes and cancers but also the protein similarities and differences of various edible seaweeds.

**Figure 3:**
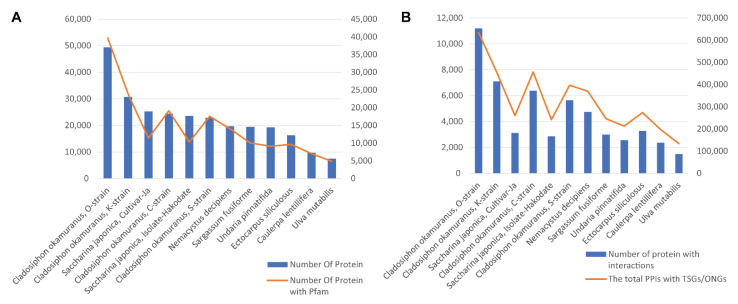
Putative interactomes summary between human cancer and 12 seaweed genome proteins. (**A**) The number of protein sequences in the 12 seaweed genomes (blue histogram); the number of proteins that can be mapped to the Pfam domain (orange broken line). (**B**) The number of proteins in the 12 seaweed genomes that putatively interact with human cancer-related proteins (blue histogram); the number of interactions with human cancer proteins in each seaweed species (orange).

Among the 12 species, the *Cladosiphon okamuranus*, O-strain, had the largest proteome, with 49,400 predicted protein sequences (Figure 3A). The other three *Cladosiphon okamuranus* strains also had relatively smaller proteome sizes, ranging from 22,921 proteins (S-strain) to 30,694 proteins (K-strain). The two cultivars of Saccharina japonica followed, with 25,245 and 23,598 proteins. The smallest proteome was obtained from the sea lettuce *Ulva mutabilis*, including 7468 proteins. The number of proteins associated with Pfam domains generally correlated with the proteome sizes (Figure 3A). However, the two *Saccharina japonica* proteomes had relatively lower ratios of proteins annotated by Pfam (0.437 and 0.452), which might imply the potential novel functions in their proteomes. In general, seaweeds with larger proteomes and more Pfam annotations tended to have more proteins interacting with cancer proteins (Figure 3B). For instance, the *C. okamuranus*, O-strain, had 11,193 proteins connected with 805 TSGs/OCGs, which were predicted to form 634,640 interactions. In contrast, *Ulva mutabilis* had only 134,389 cancer-related interactions associated with 1498 proteins. 

**Figure 4:**
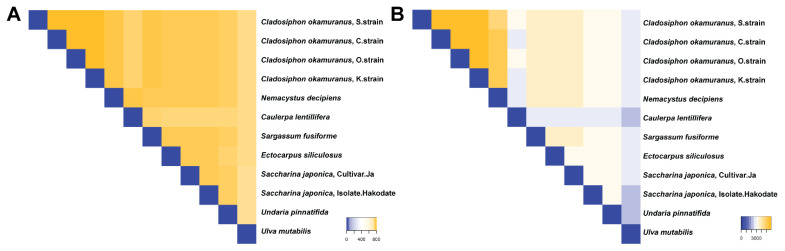
Summary of overlapping features among the 12 seaweed putative interactomes based on (**A**) cancer-related genes and (**B**) Pfam protein domains.

By comparing the 12 putative interactomes, we identified how many proteins were shared across species, including the types of protein Pfam domains (Figure 4). Overall, cancer proteins (TSG/OCG partners) involved in each putative interactome were very similar, ranging from 735 to 798 (av. 767.636, standard deviation = 16.727) (Figure 4A). In contrast, the Pfam domains involved in each putative interactome had more variation, ranging from 2345 to 5919 (av. 3541.55, standard deviation = 912.343) (Figure 4B). For instance, *C. okamuranus*, O-strain, and *C. okamuranus*, K-strain, had 5919 Pfam domains in common. In summary, the interacting TSG/OCG partners for the 12 putative interactomes were similar, while the Pfam domains to bridge the interaction may be different, depending on the seaweed. 

We defined a TSG/OCG ratio according to the number of TSG and OCG involved in each Pfam domain. With a higher ratio, a protein domain tends to function as a TSG. For example, if the ratio is equal to 1, the specific protein domain has only TSGs annotated and no association with any OCGs. On the contrary, if the ratio equals 0, these domains are only found in OCG and have not appeared in TSGs. The ratios between 0 and 1 were grouped as Pfam domains with dual roles in cancer development. In this way, we have three groups of Pfam domains: TSG-specific, OCG-specific, and dual role domains. We further ranked those Pfam domains with the most abundant interactions by counting how many interactions were involved for each Pfam domain. 

As shown in Table 2, the top twenty Pfam domains with the most abundant interactions were categorised based on domain function. Strikingly, we found that most of the top 20 ranked Pfam domains (13 out of 20) belong to dual role domains. These domains are more like molecular switches to determine cellular destiny. There were two TSG-specific (Leucine-Rich Repeat and Zinc finger, C3HC4 type) and one OCG-specific domain (Ankyrin repeat). The ratios of TSGs over OCGs for those sixteen domains ranged from 0 (pure OCG) to 1 (pure TSG). The average ratio was 0.632, which is higher than 0.5, which means that most of these domains tend to have tumour-suppressive functions.

Since our results implied that those dual role domains have more TSG functions and fewer OCG functions, we used the protein kinase domain as an example. Because of some well-known gain-of-function mutations in kinases [33], kinases have been primarily defined as oncogenes. Therefore, many kinase inhibitors are used as targeted drugs in cancer treatments. However, the outcomes of PKC inhibitors-based drugs have been disappointing in the last 30+ years. High-throughput cancer genomic data have accumulated more evidence that loss-of-function mutations in kinases could function as tumour suppressors [34]. Our data found 2,489,852 protein–protein interactions associated with 24,145 seaweed proteins. 

**Figure 5:**
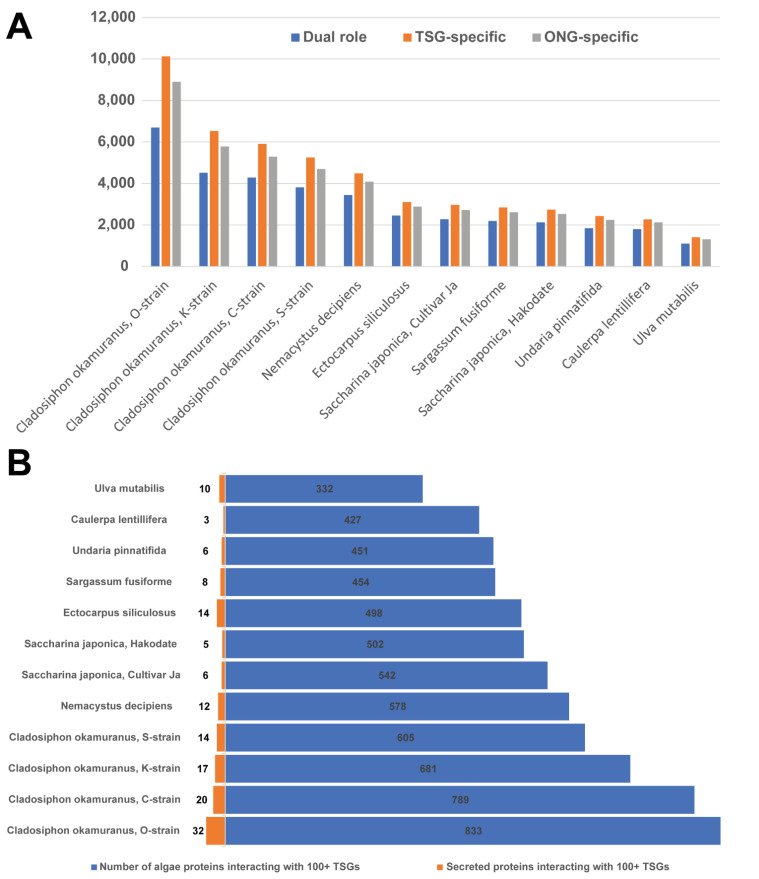
Overview of interactions between seaweed proteins and cancer proteins. (**A**) The number of interacting seaweed proteins with TSG-specific, OCG-specific, and dual-role genes. (**B**) The number of seaweed proteins interacting with 100 or more TSGs and the number of secreted proteins interacting with 100 or more TSGs.

To explore the potential effects of the interactions, we counted seaweed protein interactions against the three cancer groups: 129 dual role genes, 674 OCGs, and 1088 TSGs (Figure 5A). Of the 3,881,014 total interactions, 269,172 (about 7%) involved dual role domains. For example, the *C. okamuranus* O-strain had 6691, 10126, and 8900 proteins interacting with both TSGs and OCGs. Since the total sum of these three numbers was less than the total number of interacting proteins in the *C. okamuranus O-strain*, some proteins may interact with both TSGs and OCGs. In general, the size of the proteomes correlated with the number of interactions across 12 seaweeds. The number of proteins interacting with TSGs and OCGs was very close in each species. Although only 129 dual role genes (only 11.9%) were found in the total number of TSGs, the numbers of proteins interacting with dual role genes are over half of that number with TSGs in all the twelve proteomes. This may confirm our previous observation, where the dual role domains tended to have more interactions with seaweed proteins. To explore the potential anti-cancer effect, we also collected 6692 proteins interacting with 100 or more TSGs (Figure 5B). By further focusing on secreted proteins, we identified 147 proteins from all the 12 seaweeds that interact with more than 100 TSGs. Since secreted proteins have important roles in cell–cell signaling, communication, growth, they may have potential nutraceutical functions for the malignant conditions.

### 2.3. Essential Cancer Proteins with the Most Abundant Interactions with Seaweed Proteins

**Figure 6:**
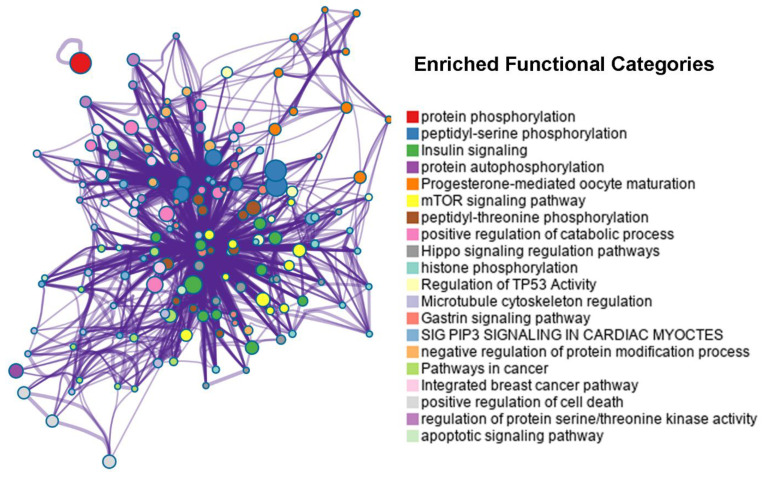
Network visualisation of enriched functional categories coloured by category cluster, where nodes that share the same colour are highly semantic similar to each other. Functional clusters are presented in different colours.

The putative interactome between the seaweed and human is important to identify the potential therapeutic targets. Since our putative interactomes mainly focused on TSGs and OCGs, we further explored the importance of these cancer proteins based on the number of interactions with seaweed proteins. Focusing on the top 50 TSGs/OCGs, we found those cancer proteins involved at least 23,720 interactions. We then conducted functional enrichment analyses to provide an overview the molecular functions (Figure 6). Of most interest were 48 human cancer proteins that are known to be involved in protein phosphorylation (Figure 6, adjusted *p*-value < 10^−76^). As one of the most well-studied post-translational modifications, protein phosphorylation is like a switch that controls many fundamental cellular processes, especially cancer-related cell growth [35]. After further analysis of those phosphorylation proteins, we found that half of them were associated with peptidyl-serine phosphorylation (24 proteins, adjusted *p*-value < 10^−39^). Another functional category of interest was insulin signalling (17 genes, adjusted *p*-value < 10^−26^). There is some sporadic evidence for an association between seaweed and human insulin. For example, in healthy postmenopausal women, serum concentrations of insulin-like growth factor 1 (IGF-1) change following seaweed supplements [36]. Recent epidemiological studies have demonstrated a link between insulin resistance and cancer with regard to cancer [37]. In addition, IGF-1 appears to play a role in tumour initiation and development in insulin-resistant patients. Furthermore, a water-soluble polysaccharide (laminarin) from the seaweed *Laminaria digitata* was found to induce apoptosis in HT-29 colon cancer cells via an insulin-like growth factor [38]. In summary, those leading interacting cancer proteins may provide some additional clues for the anti-cancer effects of seaweed. There might be more nutraceutical targets such as phosphorylation and insulin signalling. 

**Figure 7:**
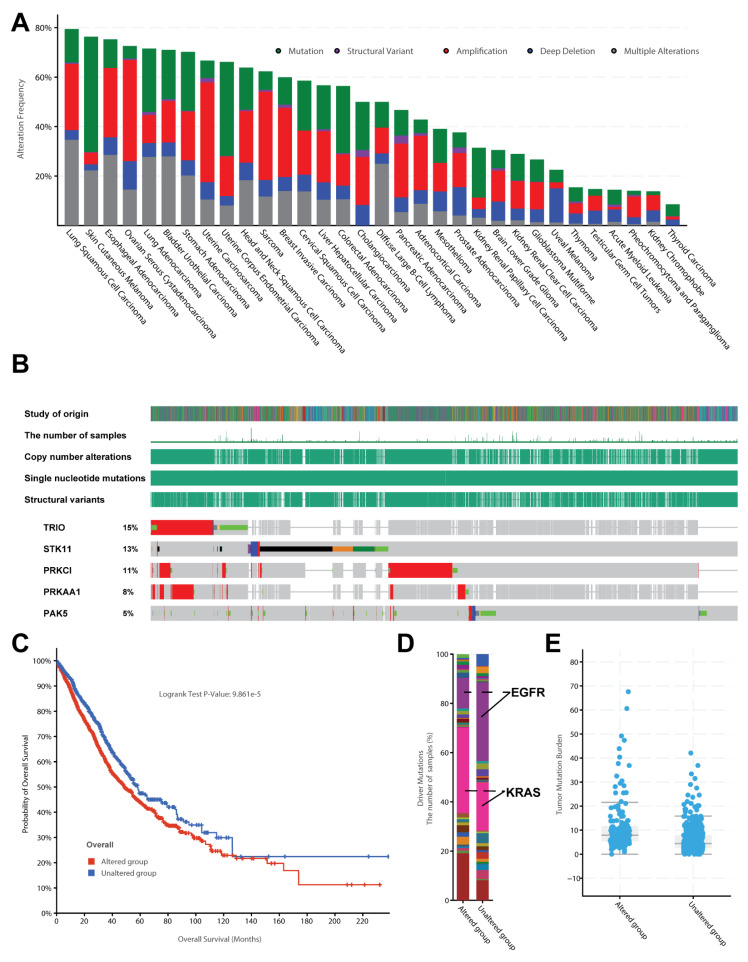
Clinical features for the top 50 cancer proteins interacting with seaweed proteins based on the TCGA cancer genomics data. (**A**) Mutational frequency among the 32 TCGA pan-cancer datasets. (**B**) The sample-based oncoprint for the top five genes mutated in thousands of lung cancer cohorts across 24 independent genomic studies. (**C**) Survival curve for the top 50 genes in thousands of lung cancer cohorts across 24 independent lung cancer genomic studies. (**D**) Driver mutations of the samples with and without mutations on the top 50 genes; KRAS mutations are in pink, while EGFR mutations are marked in purple. (**E**) Mutation burden of patient groups with and without mutations on the top 50 genes.

To link these key genes with public cancer genomics data, we explored the mutational and clinical features of those top-ranked cancer proteins using large-scale cancer genomic data. We found there were a large number of somatic mutations in various cancers (Figure 7A). For example, lung cancer, which ranks the highest mutated cancer type, has a mutational rate close to 80% (Figure 7B). By further focusing on other independent lung cancer datasets, we confirmed that the mutation rate of these 50 cancer proteins is relatively higher in lung cancer (Figure 7B, Table S2). For example, the top five genes with mutation frequency were *TRIO* (15%), *STK11* (13%), *PPKCI* (11%), *PRKAA1* (8%), and *PAK5* (5%). The most important thing is that these top mutated genes can significantly affect the cancer prognostic survival time (Figure 7C, *p*-value is less than 9.861 × 10^−5^). We further divided the patients into two groups by analysing the clinical data of those lung cancer patients. One group was patients with somatic mutations; the cancer genomic data of the other group of patients did not involve any mutations in these 50 genes. Interestingly, these two groups of patients had significant differences in the composition of driver genes (Figure 7D). For example, the mutation group often used KRAS as the primary driver gene.

In contrast, the non-mutation group mainly used EGFR as the primary driver gene to promote the occurrence and development of cancer. In addition, the two groups of patients also had significant differences in tumour mutation burden (TMB) (Figure 7E). TMB is generally calculated as the number of mutations per megabase (mut/Mb) [39]. The patients with high TMB of 10 mut/Mb or greater were revealed to respond to immune checkpoint inhibitors. In this way, these drugs could help activate the immune system to recognise cancer cells better. On the contrary, the low TMB cases had fewer mutations and decreased the possibility to activate the immune system.

## 3. Discussion

As the global population grows and life expectancy increases, cancer will become more common, and the global cancer burden will further increase, especially in low- and middle-income countries. However, many deaths caused by cancer can be avoided. About one-third of cancer deaths are due to five major behavioural and dietary risk factors: high body mass index, low intake of fruits and vegetables, lack of exercise, tobacco use, and alcohol consumption. The nutraceutical approach promises to maintain human well-being and prevent cancer [40]. As well, products derived from seaweeds have the potential to be used as therapeutics for the benefit of diagnosed cancer patients. 

Although many seaweeds have anti-cancer effects, the mechanism of action at the molecular level, such as protein–protein interaction, is largely unknown. However, this information could help inform potential targets for nutritional or pharmacological studies. Different types of secondary metabolites (e.g., terpenoids, alkaloids, polyketides, and peptides) have been isolated from marine algae and found to have a wide range of biological activities such as antimicrobial, antitumor, anti-inflammatory, antioxidant, antiviral, antimalarial, antitubercular, and anti-aging [41,42]. In addition, thousands of compounds have been investigated regarding their competitive role to protein–protein interaction, with encouraging anti-cancer effects. For example, maraviroc is a CCR5/gp120 interaction inhibitor, which are currently on the market as anti-HIV medication [43]. Several anti-cancer drugs have also entered clinical trials, demonstrating the utility of the PPI targeting strategy in cancer [43]. Our objective in this study was to establish a genome with deduced proteome resource for edible seaweeds, which was used to explore the putative interactomes between seaweed and human cancer proteins. We expected some seaweed proteins to affect cancer initiation and progression by interacting with crucial cancer proteins, such as those derived from tumour suppressors and oncogenes. As the first comprehensive interactomes of its type, our data not only provided a reusable resource but also presented a hypothesis report on the prominent features of interactomes between seaweeds and human cancers. For instance, we found that seaweed protein kinase was one of the most abundant binding domains to initiate the interactions. 

There has been a lack of systematic comparison of the similarities and differences of the anti-cancer effects among seaweeds. Our work identified common interacting protein domains found in various seaweeds. At the same time, it also helped to elucidate some species-specific interactions. Based on our observation, the 12 seaweed–cancer interactomes consist of a similar set of cancer proteins. However, a massive variation of seaweed proteins interact with cancer proteins. With more edible seaweeds identified, we could expect more discoveries about the anti-cancer effects such as immune-boosting [44]. Regarding the inter-species interactome, the proof-of-concept research has been conducted between yeast and human [45]. The main conclusion is that the yeast and human proteomes are still capable of building a biophysical network with features similar to intraspecies networks after a billion years of evolutionary difference. 

The biological availability of nutritional and functional food components is one of the key assumptions for this study. Secreted proteins could circulate throughout the human body and, therefore, are the therapeutics with access to most organs and tissues [46]. Many secreted proteins are the key regulators of cellular energy supply and macronutrient balance such as hormone, cytokines, growth factors, and chemokines. For instance, a 28-amino-acid peptide secreted by *Pseudomonas aeruginosa* is a promising anti-cancer therapeutic option in adult and paediatric solid tumours [47]. Through our analysis, we identified 50 human proteins with at least 23,720 interactions with seaweed proteins. Among them, 17 human proteins were involved in insulin signalling. Due to the importance of insulin signalling, these interacting seaweed partners may constitute a large proportion of potential therapeutic agents or be targets for developing therapeutic antibodies. We believe a complete understanding of the size and composition of this group of proteins will provide effective screening methods specifically for seaweed proteins with anti-cancer effects. Knowledge of seaweed protein interactions with human proteins may also benefit our ability to mitigate other diseases, such as cardiovascular disease [48]. Therefore, our computational approach may also be applied to cardiovascular disease to identify the potential nutraceuticals. In addition, similar research could explore regulatory interactions between plants and human cancers such as plant microRNAs [49]. 

This research has used our newly established edible seaweed resource to predict protein interactomes between seaweed and human; however, there still exists large knowledge gaps in translating the static genome information into dynamic biological interaction. Since the anti-cancer effects of seaweeds have, to date, been primarily associated with secondary metabolite activity, the standardised proteome data will be helpful in exploring a broader systems biology analysis that includes secondary metabolites with potential anti-cancer effects. 

It should be noted that seaweeds may also contain natural or environmentally derived products that are detrimental to human health. For example, seaweeds have an incredible ability to absorb trace nutrients from seawater and store them in its tissues [14]. This is one of the reasons edible seaweeds are so nutritious, but it also means they can absorb toxins such as lead, cadmium, and arsenic. These heavy metals are found in abundance in the world’s oceans due to geological and human sources. Recent heavy metal and other contaminant testing of seaweeds revealed that most seaweeds contain lead, cadmium, and arsenic in their tissues [50]. The majority of seaweed products on the market today almost certainly contain traces of one or more of these metals. Levels may differ between species, but they also differ depending on environmental factors such as temperature, salinity, and pH. Although our original goal is to explore the potential anti-cancer effects of seaweed, our data could also be useful to monitor the potential carcinogenic effect of seaweeds.

## 4. Materials and Methods

### 4.1. Gene and Protein Sequences from 12 Seaweed Genomes

We downloaded the genome assembly in Fasta format for 12 seaweeds with genomic DNA assemblies available. By following the link in their associated manuscript, we further downloaded eight proteomes, which contained all proteins sequences in Fasta format. However, four genomes did not provide gene regions and protein sequences, and therefore, a gene prediction pipeline was performed to extract putative protein sequences from the genome assembly. 

For gene prediction, we used the ab initio program Augustus (Version 3.4.0) [51]. To avoid any false-positive gene structure, we repeated masking based on the genome assembly [52]. To improve gene prediction accuracy, we prepared a training set for each specie by using Benchmarking Universal Single-Copy Orthologs (BUSCO, version 5.2.2) [53]. In general, BUSCO analysis collected a set of BUSCO core genes based on evolutionarily conserved sequences, which were used as a training dataset for gene prediction tools. In practice, the trained gene set can provide the genome features such as the length distribution of exons and the average number of exons per gene. The gene prediction tool Augustus can utilise those genome features to optimise the gene structures. To build the interactome using Pfam domains, we performed HMMersearch [54] against the Pfam database (Release 34.0) to associate the Pfam entries with all 12 proteomes. 

### 4.2. Database Construciton and Web Page Design

The resulting database has a convenient web-based interface to facilitate textual searches. All the interactome are downloadable for advanced bioinformatics data mining. The database was implement using MYSQL system and Perl CGI scripts. 

### 4.3. Domain–Domain Interaction-Based Interactome with Tumor Suppressors and Oncogenes 

To explore the protein–protein interactions with cancer proteins at the genome level, we proposed a computational framework by starting with human genes of known functions as TSGs and OCGs. This study collected cancer genes with literature evidence from TSGene 2.0 [2] and ONGene [3]. In TSGene 2.0, a total of 1217 human genes were downloaded. For ONGene, there were 803 human OCGs. Between these two lists, we found 129 shared genes, which were cancer genes with dual roles. In total, we obtained a non-redundant list of 1819 cancer genes. We found that 1560 TSGs and OCGs were annotated with Pfam domains by downloading their annotation from the ENSEMBL database [55].

One of the most common mechanisms of protein functioning is through interacting with other proteins in the cells. The protein–protein interactions are accomplished by functional domains interacting with other protein domains physically. Therefore, high-quality protein–protein interaction relationships could be summarised from biological pathway databases, such as KEGG and Reactome databases. Thus, the domain–domain interaction consists of non-redundant protein–domain interactions derived from the well-studied protein–protein interactions. In this study, the putative protein–protein interactions between the seaweeds and cancer genomes were based on protein domain–domain interactions. 

Taking the *Cladosiphon okamuranus* (O-strain) genome as an example, we constructed a protein–domain interaction network linking *C. okamuranus* (O-strain) proteins to human cancer proteins. Since we already assigned the protein-coding genes to protein domains, the high confidence domain–domain interactions from the database DOMINE were used to link the protein domains from the algal genomes to the protein domains in human cancer genes [32].

To predict the potentially secreted proteins, we utilised a computational approach by a combination of SignalP [56] and TMHMM [57]. Specifically, SignalP (version 5.0) used a deep neural network-based to predict all the possible secreting peptides with protein sequences as input. TMHMM (version 2.0) was employed to determine if a protein has transmembrane domains. By subtracting those transmembrane proteins from the predicted secreting peptides, we identified a total of 147 proteins with over hundreds of interactions with cancer proteins across all the twelve species (File S1). 

### 4.4. Cancer Genomic Data Integration and Clinical Applications 

For those leading cancer genes with the most abundant interactions with seaweed genomes, we further investigate the enriched functional categories using MetaScape [58]. In brief, the hypergeometric model was used to calculate the statistical significance of enriched annotations using the protein-coding genes in the human genome as a background. The corrected P-values for enriched annotations were calculated in MetaScape using the Benjamini–Hochberg multiple correction method. Finally, for each gene set, enriched annotations with corrected *p*-values of 0.01 were identified as over-representative annotations.

Based on the existing public platform cBio Portal [59], we determined whether the genetic mutations from the leading fifty cancer genes have clinical significance in multiple cancers. To this aim, we firstly explored the mutations frequency of those cancer genes in The Cancer Genome Atlas (TCGA) pan-cancer dataset. The summary of the pan-cancer data informed us which cancer type has the highest mutation frequency. We then focused on cancer with the highest mutation frequency and validated the results in multiple independent cancer genomics datasets for the cancer type. In our case, we focused on lung cancers. Finally, survival analyses were used to determine a statistically significant difference in survival between patients with these genetic mutations and patients without mutations in multiple lung cancer datasets. The log-rank test was used to determine whether there was no difference in survival between two patient groups. The test compares the entire survival time of patients with and without genetic mutations in specific genes. 

## 5. Conclusions

In this study, we have shared 12 genomes and proteomes of edible seaweed via a user-friendly web interface. To provide evidence for potential anti-cancer proteins, we also constructed putative interactomes constituting seaweeds and human cancer proteins. The computational results highlighted potential protein families/domains with anti-cancer effects. We found that dual role domains were the leading functional blocks that bridge the interactions, which include secreted seaweed protein interacting partners. In addition, a high number of cancer proteins that interacted with seaweed proteins were associated patient survival. In conclusion, our online portal may provide a data infrastructure to explore functional interactions between oncogenesis and anti-cancer effects in those seaweed genomes.

## Figures and Tables

**Table 1:** Summary of 12 seaweeds with publicly available assemblies.

Species	Anti-Cancer	Publication Title
*Cladosiphon okamuranus*, O-strain	Colorectal cancer [24]	Comparative genomics of four strains of the edible brown alga, Cladosiphon okamuranus [19]
*Cladosiphon okamuranus*, K-strain	Colorectal cancer [24]	Comparative genomics of four strains of the edible brown alga, Cladosiphon okamuranus [19]
*Cladosiphon okamuranus*, C-strain	Colorectal cancer [24]	Comparative genomics of four strains of the edible brown alga, Cladosiphon okamuranus [19]
*Cladosiphon okamuranus*, S-strain	Colorectal cancer [24]	A draft genome of the brown alga, Cladosiphon okamuranus, S-strain: a platform for future studies of ‘mozuku’ biology [18]
*Saccharina japonica*, Cultivar-Ja	Colorectal cancer [25]	Saccharina genomes provide novel insight into kelp biology [22]
*Saccharina japonica*, Isolate-Hakodate		Saccharina genomes provide novel insight into kelp biology [22]
*Nemacystus decipiens*	Cervical, Kidney, Breast [26] and Colorectal cancers [27]	Draft genome of the brown alga, Nemacystus decipiens, Onna-1 strain: Fusion of genes involved in the sulfated fucan biosynthesis pathway [17]
*Sargassum fusiforme*	Colorectal cancers [28]	First Draft Genome Assembly of the Seaweed Sargassum fusiforme [21]
*Undaria pinnatifida*	Breast cancer [26]	First Genome of the Brown Alga Undaria pinnatifida: Chromosome-Level Assembly Using PacBio and Hi-C Technologies [20]
*Ectocarpus siliculosus*	Unknown	The Ectocarpus genome and the independent evolution of multicellularity in brown algae [15]
*Caulerpa lentillifera*	Glioblastoma [29]	A siphonous macroalgal genome suggests convergent functions of homeobox genes in algae and land plants [30]
*Ulva mutabilis*	Unknown	Insights into the Evolution of Multicellularity from the Sea Lettuce Genome [16]

**Table 2:** The top twenty Pfam domains with the most interactions across 12 seaweeds.

PfamID	Name	Description	Category	Dual Role Ratio ^1^
PF00069	Pkinase	Protein kinase domain	Dual role	0.542857
PF00400	WD40	WD domain, G-beta repeat	Dual role	0.722222
PF00515	TPR_1	Tetratricopeptide repeat	Unknown	Not available
PF07714	Pkinase_Tyr	Protein tyrosine kinase	Dual role	0.315068
PF00271	Helicase_C	Helicase conserved C-terminal domain	Dual role	0.833333
PF00023	Ank	Ankyrin repeat	OCG	0
PF00072	Response_reg	Response regulator receiver domain	Unknown	Not available
PF00270	DEAD	DEAD/DEAH box helicase	Dual role	0.75
PF00076	RRM_1	RNA recognition motif	Dual role	0.357143
PF00560	LRR_1	Leucine Rich Repeat	TSG	1
PF00595	PDZ	PDZ domain (also known as DHR or GLGF)	Dual role	0.8
PF00071	Ras	Ras family	Dual role	0.4
PF00169	PH	PH domain	Dual role	0.375
PF00070	Pyr_redox	Pyridine nucleotide-disulphide oxidoreductase	Unknown	Not available
PF00571	CBS	CBS domain	Unknown	Not available
PF00168	C2	C2 domain	Dual role	0.705882
PF00989	PAS	PAS fold	Dual role	0.857143
PF00097	zf-C3HC4	Zinc finger, C3HC4 type (RING finger)	TSG	1
PF00249	Myb_DNA-binding	Myb-like DNA-binding domain	Dual role	0.666667
PF00149	Metallophos	Calcineurin-like phosphoesterase	Dual role	0.8

^1^ Dual role ratio = number of associated tumour suppressors/total number of associated tumour suppressors and oncogenes.

## Data Availability

The genome assemblies used were from public databases including OIST genome portal and NCBI genome data portal (GCA_000310025.1, GCA_000978595.1, GCA_012845835.1, and GCA_900538255.1).

## Supplementary Materials

The following are available online at https://www.mdpi.com/article/10.3390/biology11101458/s1, Table S1: The 299 Pfam domains with dual roles (tumour suppressive and oncogenic). Table S2: The mutational frequency of the top fifty cancer genes with the most abundant interactions across the TCGA pan-cancer dataset. File S1: The sequences of 147 secreted algae proteins interacting with 100 or more tumour suppressors.
